# Clinical impact of left atrial enlargement in Korean patients with atrial fibrillation

**DOI:** 10.1038/s41598-021-03266-z

**Published:** 2021-12-10

**Authors:** Min Soo Cho, Hyoung-Seob Park, Myung-Jin Cha, So-Ryoung Lee, Jin-Kyu Park, Tae-Hoon Kim, Jung Myung Lee, Junbeom Park, Hyung Wook Park, Ki-Woon Kang, Jaemin Shim, Jae-Sun Uhm, Jin-Bae Kim, Changsoo Kim, Young Soo Lee, Eue-Keun Choi, Boyoung Joung, Jun Kim

**Affiliations:** 1 Division of Cardiology, Asan Medical Center, University of Ulsan College of Medicine, 88 Olympic-ro, 43-gil, Songpa-gu, Seoul, 05505 Republic of Korea; 2 Division of Cardiology, Department of Internal Medicine, Cardiovascular Center, Keimyung University Dongsan Hospital, Keimyung University College of Medicine, Daegu, Korea; 3 Department of Internal Medicine, Seoul National University Hospital, Seoul, Republic of Korea; 4 Department of Cardiology, Hanyang University Seoul Hospital, Seoul, Republic of Korea; 5 Division of Cardiology, Department of Internal Medicine, Severance Cardiovascular Hospital, Yonsei University College of Medicine, 50-1 Yonsei-ro Seodaemun-gu, Seoul, 03722 Republic of Korea; 6 Division of Cardiology, Department of Internal Medicine, Kyung Hee University Hospital, Kyung Hee University, Seoul, Republic of Korea; 7 Department of Cardiology, School of Medicine, Ewha Womans University, Seoul, Republic of Korea; 8 Department of Cardiology, Chonnam National University Hospital, Chonnam National University School of Medicine, Gwangju, Republic of Korea; 9 Division of Cardiology, Eulji University Hospital, Daejeon, Republic of Korea; 10 Division of Cardiology, Department of Internal Medicine, Korea University Medical Center, Seoul, Republic of Korea; 11 Department of Preventive Medicine, Institute of Human Complexity and Systems Science, Yonsei University College of Medicine, Seoul, Korea; 12 Division of Cardiology, Department of Internal Medicine, Daegu Catholic University Medical Center, Daegu, Republic of Korea

**Keywords:** Cardiology, Medical research

## Abstract

We sought to evaluate the clinical implication of LAE based on left atrial anterior–posterior (LA AP) dimension or LA volume index (LAVI) in Korean patients with atrial fibrillation (AF). We enrolled 8159 AF patients from the CODE-AF registry. The primary outcome was rate of stroke or systemic embolism (SSE). The prevalence of mild, moderate, and severe LAE by LA AP dimension was 30.6%, 18.5%, and 21.4%, and by LAVI (available in 5808 patients) was 15.7%, 12.5% and 37.8%, respectively. Compared with no or mild LAE, patients with significant LAE (moderate to severe LAE, n = 3258, 39.9%) were associated with a higher rate of SSE (2.5% vs. 1.4%, *P* = 0.001). Multivariable analysis suggested presence of significant LAE by LA AP dimension was associated with a higher risk of SSE in the overall population (HR 1.57, 95% CI: 1.14–2.17, *P* = 0.005) and in patients using anticoagulants (n = 5836, HR 1.79, 95% CI: 1.23–2.63, *P* = 0.002). Patients with significant LAE by LAVI were also at higher risk of SSE (HR 1.58, 95% CI: 1.09–2.29, *P* = 0.017). In conclusion, significant LAE by LA dimension or LAVI was present in 39.9% and 50.2% of AF patients, respectively, and was associated with a higher rate of SSE.

## Introduction

The burden of atrial fibrillation (AF) and its associated complications is continuously growing with the aging of the global population^1^. The pathogenesis of AF is closely associated with left atrial (LA) remodelling and has recently been regarded as one of the clinical presentations of LA myopathy^2^. The pathologic change in LA remodelling is associated with AF perpetuation and AF-related complications^3,4^. Although there is currently no definitive method to evaluate the extent of LA remodelling or LA myopathy, LA enlargement (LAE) using echocardiography is a simple surrogate predictor of AF, and its complications^5–8^.

LA anterior–posterior (AP) dimension, measured in parasternal long-axis view of a transthoracic echocardiogram, is associated with adverse clinical events^9–11^. Despite measuring only one dimension of the LA geometry, it has excellent reproducibility. The LA volume index (LAVI) enables a more accurate assessment of LA remodelling, volume, and AF-related atrial substrate^12,13^. However, the clinical implications of LAE in patients with AF (AF) remains under-evaluated. Furthermore, the impact of LAE on long-term patient outcomes after introducing nonvitamin K antagonist anticoagulants (NOACs) has not yet been defined. Therefore, we have evaluated the prevalence, correlations, and prognostic implications of the LAE by AP dimension or LAVI in Korean patients with AF using data from the nationwide prospective COmparison study of Drugs for symptom control and complication prEvention of AF (CODE-AF) registry.

## Methods

### The CODE-AF registry

The CODE-AF registry is a prospective, multicenter, observational study that has included patients with AF in 18 tertiary centers across Korea since June 2016. The registry evaluates the characteristics, interventions, and outcomes of Korean patients with AF in the real-world setting. Detailed information on the study concept and design have been published previously (Supplemental material)^14^. Patients aged ≥ 19 years old who had been diagnosed with AF, and provided informed consent were eligible for this study. Data from each site were collected and entered into a web-based case report form in the Clinical Data Management System (iCReaT) of the Korea National Institute of Health. The patient's demographics, medical history, signs, symptoms, laboratory test results, electrocardiogram, echocardiography, medications, and hospital course were available in January 2020.

This study conformed to the ethical guidelines of the Declaration of Helsinki. The study protocol was approved by the ethics committee/institutional review board of each participating hospital, and the study was registered at ClinicalTrials.gov (NCT02786095, Date of registration 30/05/2016). The Ethics Committees of all 18 tertiary centers include followings; Asan Medical Center, Severance Hospital, Seoul National University Hospital, Korea University Medical Center, Daegu Catholic University Medical Center, Ewha Womans University Medical Center, Daejeon Eulji University Hospital, Kyung Hee University Hospital, Hanyang University Seoul Hospital, Chonnam National University Hospital, Inha University Hospital, Gangnam Severance Hospital, Samsung Medical Center, CHA Bundang Medical Center, Seoul St. Mary’s Hospital, Seoul National University Bundang Hospital, Keimyung University Dongsan Medical Center, Dong-a University Hospital. No patients or public were involved in any aspect of the study design, implementation of the study or the interpretation and writing of the results.

### Study population

Of the 10,868 patients enrolled in the CODE-AF registry at the time of the study, the following were excluded from the analysis: those who (1) had been diagnosed with mitral stenosis or had undergone prior valve surgery, (2) did not have transthoracic echocardiography data available, (3) did not have LA AP dimension data available, or (4) did not have a complete follow-up data or significant missing data. The data on demographic characteristics, baseline evaluations, medications, and study outcomes were derived from the registry dataset.

### Echocardiographic assessment for the left atrium

The LAE was measured at each center according to the general guideline recommendations^12^. LA AP dimension was measured in the parasternal long axis view at the level of the aortic sinuses by using the edge-to-edge measurement. The degree of LAE was classified according to the LA A-P dimension in males and females, being mild (≥ 41 mm vs. ≥ 39 mm, respectively), moderate (≥ 47 mm vs. ≥ 43 mm, respectively), and severe (≥ 52 mm vs. ≥ 47 mm, respectively). LA volume was assessed using 2D volumetric measurements based on the tracings of the blood–tissue interface on apical four- and two-chamber views. LAVI was calculated by dividing the measured LA volume by body surface area. The degree of LAE was also classified according to the LAVI (mild, > 34 mL/m^2^; moderate, ≥ 42 mL/m^2^; severe, ≥ 48 mL/m^2^). Significant LAE was defined as LAE of at least moderate degree either by LA AP dimension or LAVI.

### Study outcomes

This study's primary outcome was new-onset thromboembolic event (ischemic stroke or systemic embolism) during follow-up. Ischemic stroke was defined as an acute episode of focal cerebral, spinal, or retinal dysfunction caused by infarction, which was confirmed by an independent neurologist based on neuroimaging procedure (computed tomographic scan or brain magnetic resonance imaging)^15^. Systemic embolism was defined as a sudden loss of perfusion in a limb or organ, based on clinical manifestations as well as imaging and functional studies. Secondary outcomes consisted of all-cause mortality, myocardial infarction, any major adverse cardio-cerebrovascular event (MACCE, composite of death, stroke, systemic embolism, and myocardial infarction), all-cause bleeding, and major bleeding. Major bleeding occurred when the following criteria were met: (1) fatal bleeding; (2) fall in hemoglobin level ≥ 2 mg/dL or transfusion of ≥ 2 packs of red blood cell; and (3) bleeding in the critical area or organ (intracranial, intraspinal, intraocular, retroperitoneal, intra-articular or pericardial, or intramuscular with compartment syndrome)^16^. Clinical outcomes were adjudicated by each center’s independent research personnel, who were blinded to the patient data.

### Statistical analysis

All statistical analyses were performed using R software (version 3.3.1; www.R-project.org; R Foundation for Statistical Computing, Vienna, Austria). Comparison of categorical variables was made using the Chi-square test, and continuous variables were made using variance with post-hoc analysis with Tukey's method or the Mann–Whitney U test as appropriate. The Pearson correlation coefficient was used to quantify the associations between continuous variables. The Kaplan–Meier method was used to calculate the unadjusted event rates and comparisons between groups were made using the log-rank test. The Cox proportional-hazards model was used to assess the relative risk of each variable on the study outcomes. In the final multivariable model, the risk of LAE for each study outcome was adjusted for the components of CHA_2_DS_2_-VASc Score (age, sex, hypertension, diabetes mellitus, stroke, congestive heart failure, and vascular disease). The log [− log (survival)] curves and partial (Schoenfeld) residuals were tested with the proportional hazard assumption. The significance of the P-value was two-sided. Values that were two-sided and P-values that were < 0.05 were considered statistically significant.

### Ethics approval

The study protocol was approved by the ethics committee/institutional review board of each participating hospital.

## Results

### Baseline characteristics and prevalence of LAE

**Figure 1:**
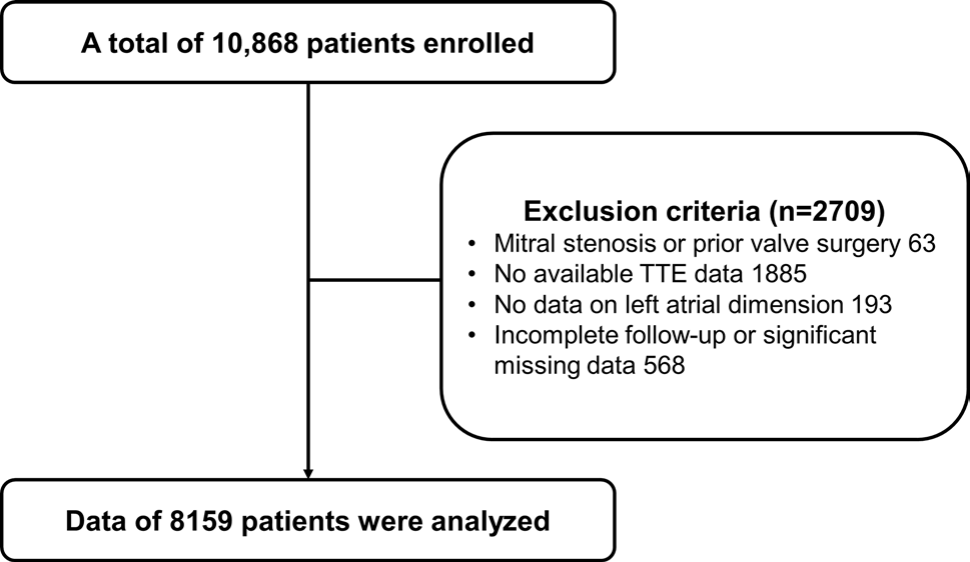
Flow-diagram.

Out of 10,868 eligible patients from the CODE-AF registry, 8,159 patients were included in the final analysis after application of the exclusion criteria (Fig. 1). Patient baseline characteristics and the results of initial evaluations are summarized in Table 1. The mean age of the population was 67.0 ± 11.5 years and 64.7% were male. The mean CHA_2_DS_2_-VASc Score was 2.7 ± 1.7 points. Mild, moderate, and severe LAE was diagnosed in 2499 (30.6%), 1511 (18.5%), and 1747 (21.4%) patients, respectively. The patients with larger LAE were characterized by a greater proportion of elders and females, and a higher prevalence of hypertension, valvular heart disease, congestive heart failure, stroke, and chronic kidney disease. Therefore, CHA_2_DS_2_-VASc Score and HAS-BLED Score were also higher in patients with greater LAE. Left ventricular ejection fraction tended to be lower, and high left ventricular diastolic filling pressure (E/E' > 15) was more prevalent in patients with a larger LA. In terms of rhythm status, persistent/permanent AF was associated with a higher degree of LAE, whereas prior RFCA was found with less significant LAE. The prescription pattern of AF medications was summarized in Table 2. In general, the rate of standard anticoagulation was used more often in the significant LAE group, whereas there were fewer antiarrhythmic prescriptions in this group.The prescriptions targeting heart failure, such as beta-blockers, ACE inhibitors/ARB, or digoxin, were higher in the significant LAE group.

**Table 1:** Baseline demographics of the study patients according to the degree of left atrial enlargement based on the left atrial anterior–posterior dimension.

Baseline characteristics	Overall	No LAE	Mild LAE	Moderate LAE	Severe LAE	*P*-value
	(n = 8159)	(n = 2402)	(n = 2499)	(n = 1511)	(n = 1747)	
Age (years)	67.0 ± 11.5	64.4 ± 13.4	66.3 ± 10.6	68.1 ± 9.9	70.7 ± 9.8	< 0.001
Male	5278 (64.7)	1559 (64.9)	1919 (76.8)	960 (63.5)	840 (48.1)	< 0.001
Body mass index (kg/m^2^)	24.7 ± 3.4	23.6 ± 3.0	24.7 ± 3.2	25.3 ± 3.4	25.5 ± 3.7	< 0.001
Hypertension	5470 (67.0)	1395 (58.1)	1648 (65.9)	1074 (71.1)	1353 (77.4)	< 0.001
Diabetes	2056 (25.2)	450 (18.7)	636 (25.5)	440 (29.1)	530 (30.3)	< 0.001
Previous MI	215 (2.6)	40 (1.7)	82 (3.3)	40 (2.6)	53 (3.0)	0.003
Valvular heart disease	853 (10.5)	99 (4.1)	193 (7.7)	189 (12.5)	372 (21.3)	< 0.001
Previous CHF	852 (10.4)	130 (5.4)	235 (9.4)	196 (13.0)	291 (16.7)	< 0.001
CIED	1425 (17.5)	494 (20.6)	516 (20.6)	245 (16.2)	170 (9.7)	< 0.001
Prior RFCA	1273 (15.6)	305 (12.7)	383 (15.3)	241 (15.9)	344 (19.7)	< 0.001
Peripheral artery disease	2907 (35.6)	811 (33.8)	865 (34.6)	544 (36.0)	687 (39.3)	0.002
Previous stroke/TIA	848 (10.4)	169 (7.0)	233 (9.3)	179 (11.8)	267 (15.3)	< 0.001
CHA_2_DS_2_-VASc score	2.7 ± 1.7	2.2 ± 1.6	2.4 ± 1.6	2.8 ± 1.6	3.4 ± 1.7	< 0.001
HAS-BLED score	1.8 ± 1.1	1.5 ± 1.1	1.7 ± 1.0	1.9 ± 1.0	2.2 ± 1.0	< 0.001
Chronic kidney disease	1462 (17.9)	279 (11.6)	415 (16.6)	309 (20.5)	459 (26.3)	< 0.001
Current smoker	723 (8.9)	234 (9.7)	249 (10.0)	140 (9.3)	100 (5.7)	< 0.001
Regular or social drinker	2358 (28.9)	714 (29.7)	836 (33.5)	436 (28.9)	372 (21.3)	< 0.001
**Electrocardiography**						
Heart rate, bpm	76.0 ± 21.4	74.6 ± 20.5	75.3 ± 23.1	76.6 ± 23.0	78.6 ± 18.1	< 0.001
QRS duration, ms	99.0 ± 25.0	99.5 ± 34.1	99.8 ± 22.4	99.2 ± 31.8	99.8 ± 24.4	0.941
QTc interval, ms	441.5 ± 86.4	436.8 ± 85.3	440.1 ± 87.8	445.7 ± 119.3	446.5 ± 37.5	< 0.001
Persistent/permanent AF	3004 (36.8)	435 (18.1)	894 (35.8)	748 (49.5)	927 (53.1)	< 0.001
Symptomatic AF	3433 (42.2)	1090 (45.4)	1036 (41.5)	577 (38.2)	735 (42.1)	< 0.001
**Echocardiography**						
LVEF, %	60.7 ± 9.6	62.2 ± 9.1	60.4 ± 9.7	59.7 ± 10.0	59.1 ± 10.7	< 0.001
LVEF < 40%	367 (4.5)	51 (2.1)	110 (4.4)	87 (5.8)	119 (6.8)	< 0.001
LA diameter, mm	43.7 ± 7.9	34.8 ± 4.2	42.4 ± 2.6	47.3 ± 2.5	54.6 ± 5.3	< 0.001
High LV filling pressure	1433 (17.6)	205 (8.5)	348 (13.9)	328 (21.7)	552 (31.6)	< 0.001

*AF* atrial fibrillation, *CHF* congestive heart failure, *CIED* cardiac implantable electrical device, *LA* left atrial, *LV* left ventricle, *LVEF* left ventricular ejection fraction, *MI* myocardial infarction, *QTc* corrected QT interval, *RFCA* radiofrequency catheter ablation, *TIA* transient ischemic attack.

Data are presented as mean ± standard deviation or number (%).

**Table 2:** Prescription pattern of medications for atrial fibrillation according to the degree of left atrial enlargement by left atrial anterior–posterior dimension.

Baseline characteristics	Overall	No LAE	Mild LAE	Moderate LAE	Severe LAE	*P*-value
	(n = 8159)	(n = 2402)	(n = 2499)	(n = 1511)	(n = 1747)	
Anticoagulation	5836 (71.5)	1368 (57.0)	1761 (70.5)	1218 (80.6)	1489 (85.2)	< 0.001
Warfarin	1264 (15.5)	224 (9.3)	350 (14.0)	265 (17.5)	425 (24.3)	< 0.001
NOAC	4752 (58.2)	1183 (49.3)	1450 (58.0)	994 (65.8)	1125 (64.4)	< 0.001
Antiplatelets	1854 (22.7)	614 (25.6)	611 (24.4)	305 (20.2)	324 (18.5)	< 0.001
Antiarrhythmics	3812 (46.7)	1414 (58.9)	1254 (50.2)	664 (43.9)	480 (27.5)	< 0.001
Class I	2726 (33.4)	1093 (45.5)	914 (36.6)	434 (28.7)	285 (16.3)	< 0.001
Class III	1261 (15.5)	363 (15.1)	398 (15.9)	268 (17.7)	232 (13.3)	< 0.001
**Others**						
Beta-blocker	4177 (51.2)	1181 (49.2)	1234 (49.4)	794 (52.5)	968 (55.4)	< 0.001
Calcium channel blocker	2263 (27.8)	545 (22.7)	710 (28.4)	460 (30.4)	548 (31.4)	< 0.001
Digoxin	571 (7.0)	74 (3.1)	132 (5.3)	128 (8.5)	237 (13.6)	< 0.001

*NOAC* non-vitamin K antagonist anticoagulant.

**Figure 2:**
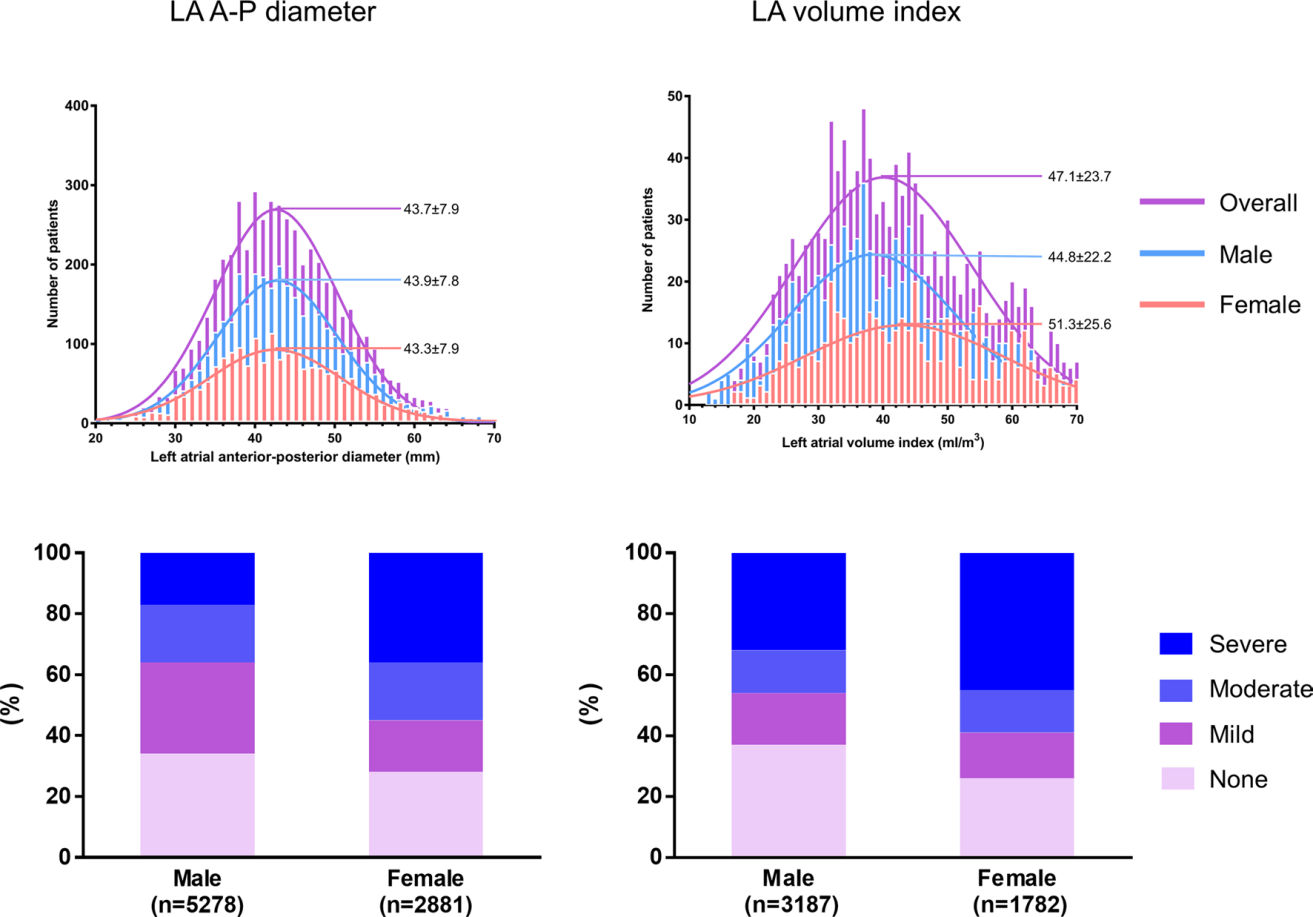
Distribution of left atrial size by left atrial anterior–posterior diameter (left) and left atrial volume index (right) in the overall population.

The overall and sex-specific distribution of LAE was depicted in Fig. 2. The mean LA AP diameter of all patients was 43.7 ± 7.9 mm and larger in male patients (43.9 ± 7.8 vs. 43.3 ± 7.9 mm). The proportion of patients with significant LAE was higher in females than in males (*P* < 0.001).

### Clinical implications of significant LAE

**Figure 3:**
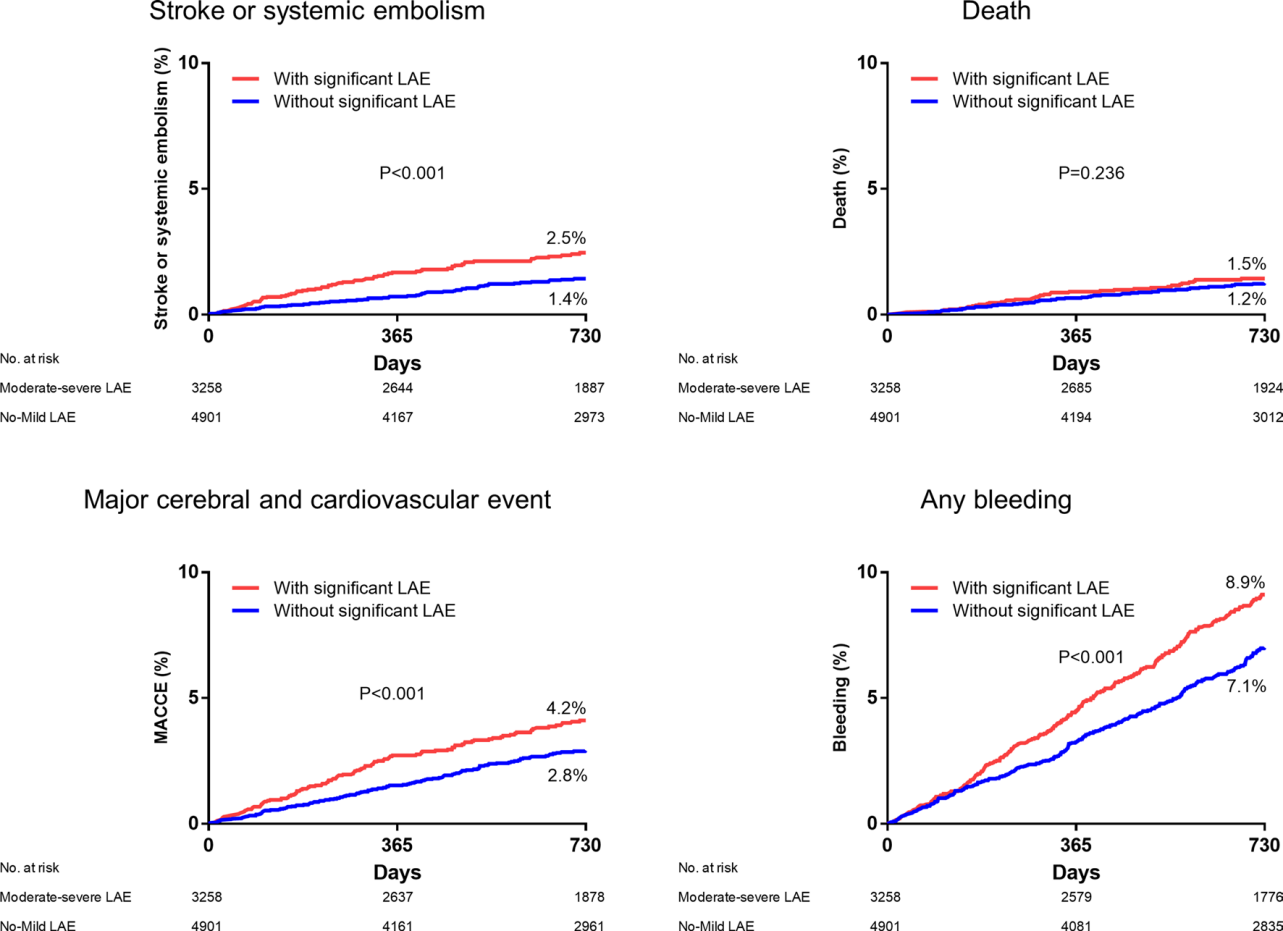
Rate of clinical outcomes according to the presence of moderate–severe left atrial enlargement.

During the mean follow-up period of 2.2 ± 0.8 years, the primary outcome of stroke or systemic embolism occurred in 2.0% of the overall population (n = 162, Table 3). The 2-year rate of the primary outcomes was significantly higher in patients with significant LAE compared with those with no or mild LAE (2.5% vs. 1.4%, *P* = 0.001, Fig. 3). Multivariable Cox regression analysis showed that the presence of significant LAE was independently associated with a higher risk for the primary outcome of stroke or systemic embolism (adjusted hazard ratio [HR] 1.57, 95% confidence interval [CI]: 1.10–2.17, *P* = 0.005, Table 4). The higher risk of LAE was consistent when the LA A-P dimension was considered as a continuous variable (per 1 mm, adjusted HR 1.03, 95% CI 1.01–1.05, *P* = 0.002). The higher rate of primary outcomes in the significant LAE group was prominent in patients with high thromboembolic risk (n = 5480, CHA_2_DS_2_-VASc score ≥ 2 in male and ≥ 3 in female, 2.9% vs. 1.9%, *P* = 0.011), but statistical significance was not reached in those with only low or borderline risk (n = 2679, CHA_2_DS_2_-VASc score of 0 to 1 in male and 1 to 2 in female, 0.9% vs. 0.7%, *P* = 0.120). This higher risk of primary outcomes was consistent when the analysis was confined to patients taking oral anticoagulation (n = 5836, adjusted HR 1.79, 95% CI: 1.23–2.63, *P* = 0.002, Table 4).

**Table 3:** Clinical outcomes according to the degree of left atrial enlargement by left atrial anterior–posterior dimension.

Baseline characteristics	No LAE	Mild LAE	Moderate LAE	Severe LAE	*P*-value
Overall population (N = 8159)	(n = 2402)	(n = 2499)	(n = 1511)	(n = 1747)	
Stroke or systemic embolism	33 (1.4)	42 (1.7)	32 (2.1)	55 (3.1)	< 0.001
Death	30 (1.2)	31 (1.2)	16 (1.1)	34 (1.9)	0.113
Myocardial infarction	4 (0.2)	15 (0.6)	7 (0.5)	5 (0.3)	0.079
MACCE	67 (2.8)	82 (3.3)	53 (3.5)	92 (5.3)	< 0.001
Major bleeding	23 (1.0)	21 (0.8)	18 (1.2)	20 (1.1)	0.658
Any bleeding	162 (6.7)	198 (7.9)	144 (9.5)	150 (8.6)	0.013
Anticoagulation population (n = 5836)	(n = 1368)	(n = 1761)	(n = 1218)	(n = 1489)	
Stroke or systemic embolism	19 (1.4)	26 (1.5)	26 (2.1)	48 (3.2)	< 0.001
Death	25 (1.8)	20 (1.1)	16 (1.3)	27 (1.8)	0.281
Myocardial infarction	3 (0.2)	10 (0.6)	4 (0.3)	4 (0.3)	0.354
MACCE	47 (3.4)	54 (3.1)	44 (3.6)	78 (5.2)	0.009
Major bleeding	18 (1.3)	17 (1.0)	16 (1.3)	17 (1.1)	0.772
Any bleeding	129 (9.4)	159 (9.0)	121 (9.9)	130 (8.7)	0.726

*MACCE* major adverse cardiac and cerebrovascular event.

Data are presented as number (%).

**Table 4:** Prognostic implication of significant left atrial enlargement on clinical outcomes.

	Crude analysis				Multivariable analysis	
	Event rate (%) at 2 years		HR (95% CI)	*P*-value	HR (95% CI)	*P*-value
	Without significant LAE	With significant LAE				
Overall population (N = 8159)	(n = 4901)	(n = 3258)				
Stroke or systemic embolism	1.4	2.5	1.78 (1.31–2.43)	< 0.001	1.57 (1.14–2.17)	0.005
Death	1.2	1.5	1.25 (0.86–1.82)	0.238	1.10 (0.75–1.63)	0.622
Myocardial infarction	0.4	0.4	0.97 (0.47–1.99)	0.935	0.90 (0.43–1.89)	0.775
MACCE	2.8	4.2	1.49 (1.19–1.88)	< 0.001	1.35 (1.06–1.71)	0.013
Major bleeding	0.9	1.2	1.33 (0.86–2.05)	0.203	1.10 (0.70–1.72)	0.685
Any bleeding	7.1	8.9	1.27 (1.09–1.48)	0.003	1.13 (0.96–1.32)	0.148
Anticoagulation population (n = 5836)	(n = 3129)	(n = 2707)				
Stroke or systemic embolism	1.4	2.6	1.94 (1.34–2.81)	< 0.001	1.79 (1.23–2.63)	0.002
Death	1.4	1.6	1.12 (0.74–1.70)	0.590	1.12 (0.73–1.73)	0.594
Myocardial infarction	0.4	0.3	0.73 (0.30–1.75)	0.474	0.71 (0.29–1.74)	0.449
MACCE	3.1	4.4	1.43 (1.10–1.86)	0.008	1.38 (1.05–1.81)	0.019
Major bleeding	1.2	1.3	1.11 (0.69–1.79)	0.661	1.04 (0.64–1.70)	0.868
Any bleeding	9.2	9.4	1.03 (0.87–1.22)	0.764	0.99 (0.83–1.18)	0.911

*MACCE* major adverse cardiac and cerebrovascular event.

*Adjusted for age, sex, hypertension, diabetes, previous stroke, heart failure, and vascular disease.

In terms of secondary outcomes, the risk of the MACCE was higher in the significant LAE group compared with no or mild LAE groups (4.2% vs. 2.8%, *P* < 0.001), and persisted even after multivariable adjustment (HR 1.35, 95% CI: 1.06–1.71, *P* = 0.013). The rate of any bleeding event was higher in the significant LAE group (8.9% vs. 7.1%, *P* = 0.003), but statistical significance was lost after multivariable adjustment (Table 4).

**Figure 4:**
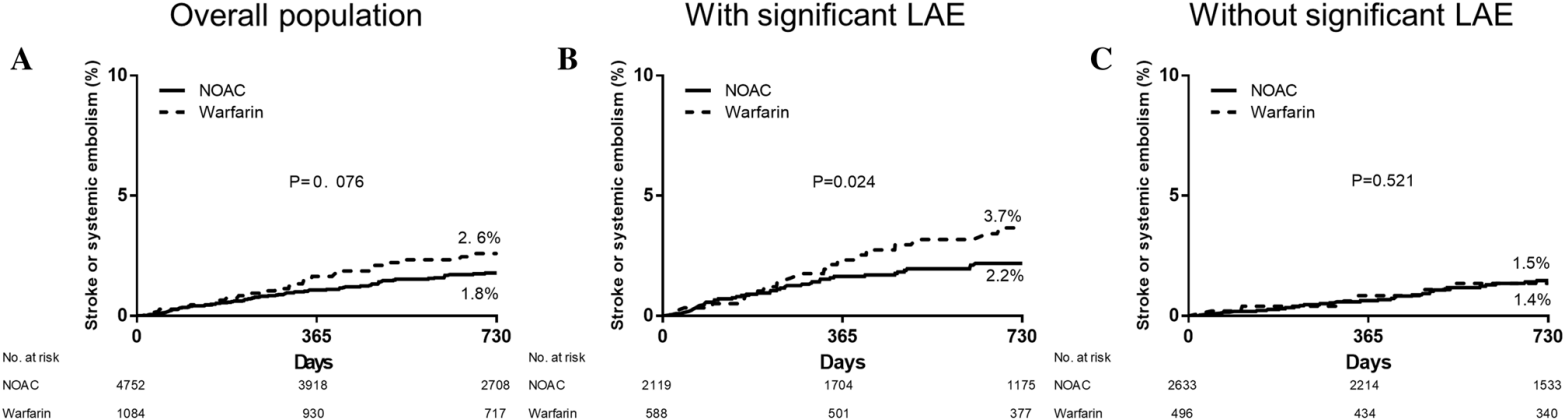
Influence of type of anticoagulation on the rate of stroke or systemic embolism stratified in (**A**) the overall population, (**B**) those with moderate to severe LAE, and (**C**) those without moderate–severe LAE.

The implications of the significant LAE on the type of anticoagulants were further analyzed. Of the 5836 patients taking oral anticoagulation, NOACs and warfarin were used in 81.4% (n = 4752) and 18.6% (n = 1084) of patients at the study entry. The crude rate of the primary study outcomes was numerically lower in patients using NOACs compared with those using warfarin at 2-year follow-up, but this was not statistically significant (2.6% vs. 1.8%, *P* = 0.076, Fig. 4). Primary outcomes were significantly different in patients with significant LAE (3.7% vs. 2.2%, *P* = 0.024), but not in those without significant LAE (1.5% vs. 1.4%, *P* = 0.521). After multivariable adjustment, the risk of primary outcomes in patients treated with NOAC was significantly lower than those using warfarin in the significant LAE group (HR 0.57, 95% CI: 0.35–0.94, *P* = 0.029), but not in the low or moderate group (HR 1.29, 95% CI: 0.54–3.08, *P* = 0.569).

### Comparison between LA dimension and LAVI

The data on LAVI were available in 71.2% (n = 5808) of the study participants. The mean LAVI was 47.1 ± 23.7 mL/m^2^ in the overall population, and significantly larger in females than in males (51.3 ± 25.6 vs. 44.8 ± 22.2, *P* < 0.001, Fig. 2). There was a modest correlation between LAVI and LA dimension (r = 0.687, *P* < 0.001). The mild, moderate, and severe LAE by LAVI was diagnosed in 912 (15.7%), 725 (12.5%), and 2194 (37.8%) patients, respectively, which was significantly different from LAE by LA A-P dimension (*P* < 0.001). Those patients with significant LAE by LAVI were at higher risk for SSE than those without (2.6% vs. 1.4%; adjusted HR 1.58, 95% CI: 1.09–2.29, *P* = 0.017, Supplementary Table). In these patients, the rate of SSE by the presence of significant LAE by LA AP dimension (2.6% vs. 1.4%, *P* < 0.001) showed a similar trend (Supplemental Fig. 1). There was no significant difference in predicting 2-years SSE by LAVI or LA A-P dimension (C statics 0.592 [0.542–0.643] for LA A-P diameter vs. 0.570 [0.524–0.616] for LAVI, *P* = 0.257). The rate of SSE after use of warfarin versus NOAC was also similar in patients with significant LAE either by LAVI (4.3% vs. 2.3%, *P* = 0.007) or LA A-P dimension (4.0% vs. 2.4%, *P* = 0.028, Supplemental Fig. 2).

## Discussion

In this nationwide, multicenter, prospective observational cohort study of patients with AF, we noted several important results. First, LAE by LA A-P diameter was found in more than half of the patients with AF. Second, a higher degree of LAE was associated with a higher number of comorbidities and persistent forms of AF. Third, significant LAE was associated with higher rates of stroke or stroke embolism, including in patients taking oral anticoagulation. Finally, the benefits of NOACs over warfarin were prominent in those with significant LAE.

The association between LAE and higher risk of thromboembolic events in patients with AF has been assessed in the previous studies. For example, Hamatani et al.^11^ found that an LA dimension > 45 mm was associated with a higher rate of stroke and systemic embolism in the Japanese general ambulatory population. Whereas Ogata et al.^9^ found higher rates of recurrent stroke in patients with AF and LAE. The LAVI is a three-dimensional measurement that accurately assesses LA volume status and structural remodelling^12^. In this prospective registry, LAVI was not available in 28.9% of patients, reflecting underutilization of major society recommendations for chamber measurement in real-world clinical practice. In contrast, data on the LA dimensions are available for most of the patients. Although the LA dimension is fundamentally a one-dimensional measurement, its main advantages include ease of measurement, smaller interobserver variability, and excellent reproducibility^12^. We noted a modest correlation between LA dimension and LAVI (r = 0.687), and the proportion of LAE by both measurements was significantly different. Specifically, woman had a higher rate of significant LAE by LAVI compared to men (58.7% vs. 45.6%, *P* < 0.001). A recent study from Li et al. demonstrated a histologic analysis of pulmonary vein tissue and demonstrated a higher degree and rate of fibrosis in women due to the upregulated expression of fibrosis-related genes and proteins^17^. There exists few clinical data regarding gender-specific assessment of LAVI, which showed conflicting results of a larger LAVI in female patients^18^, or no gender differences^19,20^. This discrepancy can be possibly explained by the difference in the imaging methods used or patient population. From a clinical perspective, the predictive performance of both measurements (LA dimension and LAVI) for SSE were not significantly different. Therefore, in the current study, we concluded that determining LAE by both LA AP dimension or LAVI is useful in predicting future SSE. Considering its ease of use, LAE by LA AP dimension is a still a useful parameter for predicting future SSE. Different clinical implications of LAE by LAVI or LA AP dimension, and their association ability to detect LA fibrosis or remodeling should be evaluated in future studies.

A higher prevalence of comorbidities and chronic forms of AF were associated with a higher degree of LAE in the patient population^21,22^. Our analysis demonstrated the independent role of LAE on thromboembolic events, even after adjustment for such comorbidities^23,24^. Specifically, this higher risk of significant LAE persisted in patients using standard anticoagulation. With the association of a higher risk of “anticoagulation failure” with significant LAE in patients already on anticoagulation, meticulous monitoring for drug adherence and need for adjunctive treatments could be considered with a personalized approach^10,25–27^. Moreover, the differential effectiveness of NOACs over warfarin by the presence of LAE was noted in our study. Compared with warfarin, NOACs demonstrated greater effectiveness in patients with significant LAE, which was not shown in those with absent or mild LAE. These results are clinically important because NOAC would be more appropriate in high-risk patients with LAE who have a high number of comorbidities. The recent study from Wu et al. demonstrated that NOACs showed were more effective than warfarin in patients with LAE. Apart from their studies, we suggest that the degree of LAE is also important, and the effectiveness of NOAC was only evident in those with moderate to severe LAE^28^. Although beyond the scope of this study, NOAC's greater effectiveness can be partially explained by the benefits of the NOACs, such as stable pharmacodynamics, fewer drug–drug or food interactions, and fewer side effects that may be exaggerated in more high-risk patients. This suggestion is supported by observations from previous studies that have shown that, compared with warfarin, NOAC's absolute risk reduction is more exaggerated in high-risk patients, such as those with hypertension or diabetes^29,30^.

There were several limitations to our analysis. Firstly, there was an inherent selection bias as a result of the observational nature of the study. Specifically, the study population consists of selected patients who were treated in tertiary centers, which could be an additional source of bias. Although LAE would be measured per the current guidelines, interobserver variability could have existed. Our multivariable model focused on the primary outcome, and therefore, very few numbers of secondary outcomes could lead to over-adjusted results after adjustment. Finally, caution should be taken when generalizing the results of this analysis because data are derived from the Korean population only.

## Conclusions

Significant LAE determined by either LA A-P dimension (> 47 mm in men; > 43 mm in women) or LAVI (> 42 mL/m^2^) was prevalent in Korean patients with AF, and significant LAE was associated with a greater risk of thromboembolic events.

## Supplementary Information

Supplementary Information 1.

Supplementary Information 2.

## Abbreviations

AF: Atrial fibrillation
CI: Confidence interval
CHA_2_DS_2_^-^VASc: Scoring system
CODE-AF: Comparison study of drugs for symptom control and complication prevention of atrial fibrillation
HR: Hazard ratio
LA AP: Left atrial anterior–posterior
LAE: Left atrial enlargement
LAVI: Left atrial volume index
MACCE: Major adverse cardio-cerebrovascular event
NOAC: Non-vitamin K oral anticoagulant
SSE: Stroke, or systemic embolism
