# Supplementary material for: Clinical impact of left atrial enlargement in Korean patients with atrial fibrillation
Source: Sci Rep. 2021 Dec 10;11:23808. doi: 10.1038/s41598-021-03266-z (PMC8664956; doi:10.1038/s41598-021-03266-z)
Supplement: Supplementary file 1 — Supplementary Information 1. [file 41598_2021_3266_MOESM1_ESM.docx]

**Comparison Study of Drugs for Symptom Control and Complication Prevention of Atrial Fibrillation (AF) (Code-AF Trial)**

**Principle investigator**

Boyoung Joung, MD, PhD

Division of Cardiology, Severance Cardiovascular Hospital, Yonsei University College of Medicine, 50-1 Yonsei-ro Seodaemun-gu, Seoul, 03722, Republic of Korea

Tel: +82-2-2228-8460

Fax: +82-2-2227-7732

E-mail: [cby6908@yuhs.ac](mailto:cby6908@yuhs.ac)

**Source of Funding:** This study was supported by a research grant from the Korean Healthcare Technology R&D project funded by the Ministry of Health & Welfare (HI15C1200, HC19C0130).

**Brief Summary**

This study is prospective Cohort study which was performed in multicenter (General Hospital) in Korea. Inclusion criteria is all patients with atrial fibrillation who visit hospital. The purpose is to analyze complication, composite outcome (all cause mortality, hospitalization, the incidence of stroke, heart failure and cardiovascular event (MACE)) according to the 1) the use of anti-arrhythmic drugs (AADs), 2) use of medication for rate control (beta blocker, calcium channel blocker and digoxin) and 3) use of anticoagulation agents (warfarin, coumadin, an NOAC)

**Rationale & background**

Atrial fibrillation (AF) is most common tachyarrhythmia met in the clinical practice. The AF related ischemic stroke, heart failure, cognitive dysfunction, and deteriorated quality of life should be prevented for improving the general health of the entire population. Therefore, the optimal medical treatments for rhythm or rate control, and stroke prevention are fundamental component of management of AF patients. However, specific data regarding optimal medical treatment for Korean patient were sparse to date. The Korean Ministry of Health & Welfare planned to establish an all-commers registry to provide supporting data for creating standard practice guidelines for Korean AF patients. The Comparison Study of Drugs for Symptom Control and Complication Prevention of Atrial Fibrillation (CODE-AF registry) is created to investigate the clinical outcomes after 1) rhythm control, 2) rate control, and 3) anticoagulation treatments in Korean patients with AF.

**Study goals and objectives**

The primary objective of the CODE-AF registry is to analyze the clinical outcomes (all-cause mortality, hospitalization, the incidence of stroke, heart failure and cardiovascular event [MACE]) according to the medications for 1) rhythm control, 2) rate control, and 3) anticoagulation in patients with AF

**Study design**

The CODE-AF registry a multi-center, prospective observational cohort study investigating clinical outcomes after medical treatment of AF

**Methodology**


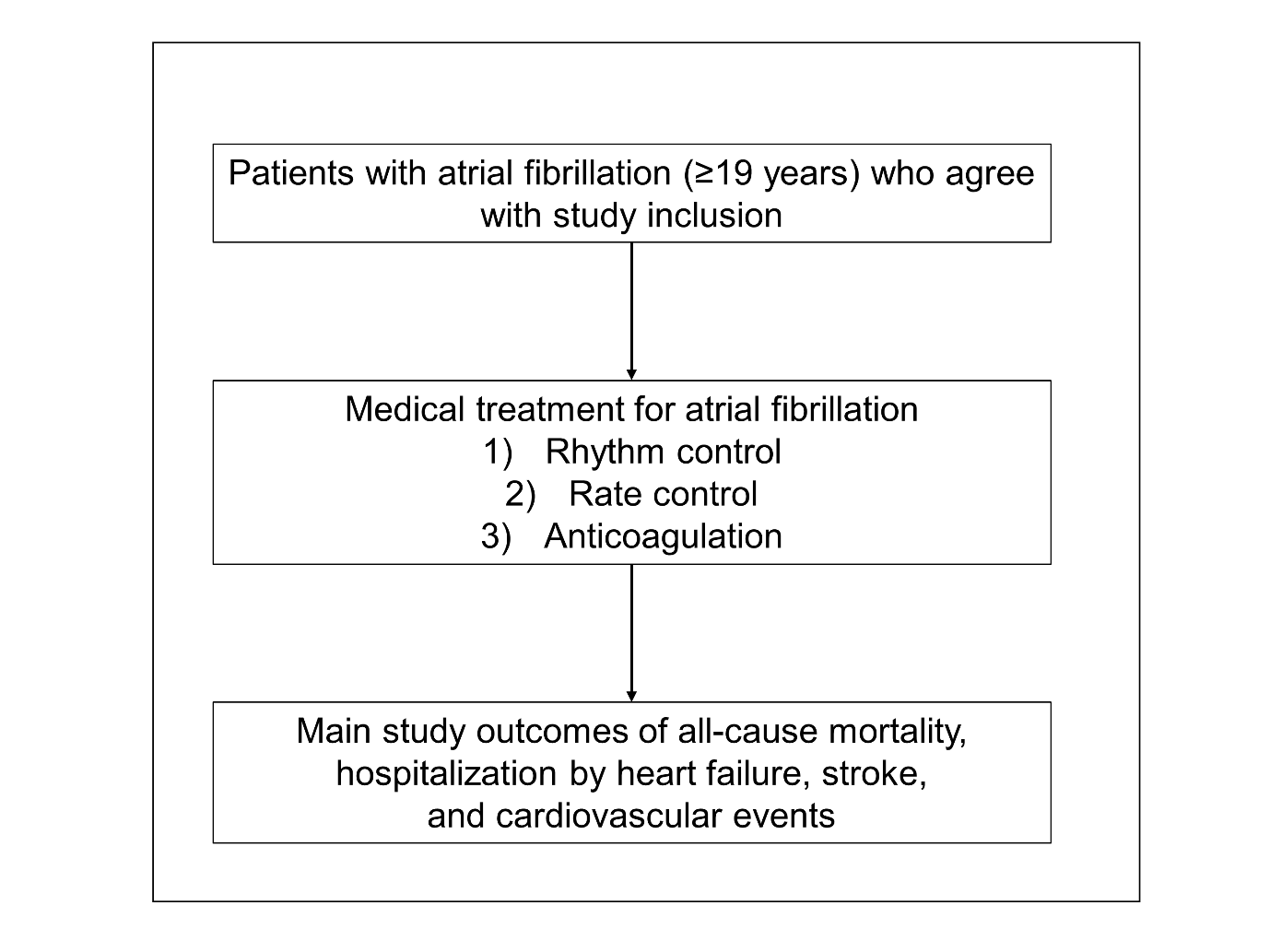


**Study population**

This prospective all-comer registry enrolls all Korean adult AF patients who agree with study inclusion

**Inclusion / Exclusion criteria**

**Inclusion**

- patients with atrial fibrillation
- patients with age more than 19
- patients who agree with study inclusion

**Exclusion**

- patients who do not agree with study inclusion
- patients with age less than 19
- Pregnancy, Breastfeeding

**Study outcomes**

**Primary study outcomes**

Main study outcomes of interests are all-cause mortality, hospitalization by heart failure, stroke, and cardiovascular events

**Secondary outcomes**

**Efficacy**

- All cause death
- Cardiovascular death
- Myocardial infarction
- Ischemic stroke
- Systemic embolism
- Rate control
- Quality of life (AFEQT questionnaire)

**Safety**

- Fatal bleeding (ISTH definition)
- Major bleeding (ISTH definition)
  - Fatal bleeding
  - Bleeding in the critical site (Intracranial, retroperitoneal, intraocular, intraspinal, intra-articular, pericardial, intramuscular with compartment syndrome)
  - Bleeding causing a fall in hemoglobin level of 2g/dL or leading to transfusion of two or more units of whole blood or red cells.
- Minor bleeding (ISTH definition)
- Intracranial hemorrhage
- Gastrointestinal hemorrhage

**Study measurement**

The baseline demographic, laboratory, electrocardiographic, and echocardiographic data are obtained at the study enrollment. Clinical follow-up will be performed at every 6 months by 36 months after the enrollment. Data collected during all follow-up visits will include death, MI, stroke, hospitalization, and all other secondary study outcomes.

**Safety considerations**

We anticipate enrolling a total of 20,000 patients. Pregnant women and children are excluded from the trial for ethical and safety concerns. Women of child-bearing potential must have a negative serum/urine pregnancy test prior to enrollment. Prior to collecting study data, the details of the study will be explained to the participant including: (1) that the study represents a research effort, (2) that participation is voluntary, and there is no penalty for withdrawal, (3) any anticipated costs to the patient for participation, (4) potential risks and benefits for participation, and (5) contact information for additional concerns. Patients are informed of the purpose of the study, the treatment alternative, the need to be available for telephone follow-up and return clinic visits at regular intervals for questionnaires and/or medical tests, and of their options to accept or refuse entry into the study without affecting their clinical care. All sources of research materials will be in the form of medical records, electrocardiograms, routine blood work, functional testing. This material will be obtained both for routine medical care as well as for research purposes.

**Follow-up**

|  | Screening | Enrollment | 6M | 12M | 18M | 24M | 30M | 36M |
| --- | --- | --- | --- | --- | --- | --- | --- | --- |
|  | Visit -1 | Visit 0 | Visit1 | Visit2 | Visit3 | Visit4 | Visit5 | Visit6 |
| Screening | X |  |  |  |  |  |  |  |
| Informed consent | X |  |  |  |  |  |  |  |
| Eligibility assessment | X |  |  |  |  |  |  |  |
| Demographics | X |  |  |  |  |  |  |  |
| Medical History | X |  |  |  |  |  |  |  |
| Baseline Physical Examination | X |  |  |  |  |  |  |  |
| CBC, Platelet | X |  |  |  |  |  |  |  |
| Electrolytes, AST/ALT, BUN/Cr | X |  |  |  |  |  |  |  |
| INR (warfarin user) | X | X | X | X | X | X | X | X |
| 12 lead ECG | X | X | X | X | X | X | X | X |
| AFEQT questionnaire | X |  |  | X |  |  |  | X |
| Echocardiography | X |  |  |  |  |  |  |  |
| Assess for concomitant medication | X | X | X | X | X | X | X | X |
| Study treatment |  | X | X | X | X | X | X | X |
| Bleeding (BARC and TIMI criteria) |  | X | X | X | X | X | X | X |
| Clinical endpoints assessment |  | X | X | X | X | X | X | X |
| Pregnancy test^2^ | X |  |  |  |  |  |  |  |

**Data management and statistical analysis**

**Sample Size Estimation**

The CODE-AF registry designed to be the evidence for making standard practice guidelines for managing Korean AF patients. The investigators concluded that enrollment of at least 20,000 patients would be required to establish a clinical registry data representative for Korean AF patients according to the expert’s consensus.

**Statistical Analysis**

Analysis of the primary and secondary study outcomes will be performed as time-to-first-event. Differences between treatment groups are evaluated by Student’s t-test for continuous variables and by the chi-square or Fisher’s exact for categorical variables. Cumulative event curves are generated by means of the Kaplan-Meier method. Statistical comparisons are based on a time-to-first-event analysis that used the Cox proportional-hazards model. Relative risks are expressed as hazard ratios with associated 95% confidence intervals and are derived from the Cox model. The level of significance for the assessment of the primary end point will be α = 0.05. We will perform all the comparisons using two-sided significance tests. All the analyses are performed with the use of R software, version 3.3.1 or higher

**Publication Policy**

Any decisions of release of results will be taken by the consensus of investigators. Manuscripts on Ancillary Studies or Subset Analyses should have the approval of the Executive Committee. The investigators significantly contributing to the study, considering both the number of patient enrollment and the study designing will have the priority in the authorships of the ancillary studies or subset analysis. The first priority of authorship on Ancillary Studies will be given to the PI or the designated investigator by the PI. The priorities of other subset studies will be given to the investigators who will enroll the largest number of patients. The investigators with the priority of authorship should be one of members in the major institutions which will enroll more than 100 study patients. Each presentation of results on behalf of the investigators should have the approval of the Executive Committee.

**Duration of the project**

A total schedule period of 8 years is expected to complete the study protocol. This estimation includes the patient’s enrollment and minimum clinical follow-up of 3 years for each patients.

**Anticipated risk**

Because the current study investigated the outcomes after standard medical treatment for atrial fibrillation, additional risk of participant will not be higher than other patients. However, specific complications after each medication could be occurred regardless of participation in the study.

**Ethics**

This study will be conducted in compliance with the protocol, the Sponsors’ standard operating procedures and local regulations where applicable, ISO 14155 guidelines and the Declaration of Helsinki.
