# Supplementary material for: Clinical impact of left atrial enlargement in Korean patients with atrial fibrillation
Source: Sci Rep. 2021 Dec 10;11:23808. doi: 10.1038/s41598-021-03266-z (PMC8664956; doi:10.1038/s41598-021-03266-z)

**Supplemental Table 1. Baseline demographics of the study patients based on left atrial volume index**

| **Baseline characteristics** | Overall | No LAE | Mild LAE | Moderate LAE | Severe LAE | P-value |
| --- | --- | --- | --- | --- | --- | --- |
|  | (n=5808) | (n=1977) | (n=912) | (n=725) | (n=2194) |  |
| Age (years) | 67.1 ±11.1 | 64.1 ±11.8 | 66.4 ±10.4 | 68.2 ±10.1 | 69.8 ±10.2 | <0.001 |
| Male | 3751 (64.6) | 1406 (71.1) | 633 (69.4) | 474 (65.4) | 1238 (56.4) | <0.001 |
| Body mass index (kg/m^2^) | 24.7 ± 3.4 | 24.7 ± 3.3 | 25.0 ± 3.4 | 24.8 ± 3.6 | 24.6 ± 3.5 | 0.470 |
| Hypertension | 4024 (69.3) | 1281 (64.8) | 628 (68.9) | 526 (72.6) | 1589 (72.4) | <0.001 |
| Diabetes | 1571 (27.0) | 485 (24.5) | 256 (28.1) | 231 (31.9) | 599 (27.3) | 0.001 |
| Previous MI | 163 (2.8) | 47 (2.4) | 33 (3.6) | 20 (2.8) | 63 (2.9) | 0.311 |
| Valvular heart disease | 687 (11.8) | 52 (2.6) | 59 (6.5) | 112 (15.4) | 464 (21.1) | <0.001 |
| Previous CHF | 538 (9.3) | 78 (3.9) | 56 (6.1) | 73 (10.1) | 331 (15.1) | <0.001 |
| CIED | 972 (16.7) | 381 (19.3) | 168 (18.4) | 118 (16.3) | 305 (13.9) | <0.001 |
| Prior RFCA | 987 (17.0) | 271 (13.7) | 138 (15.1) | 136 (18.8) | 442 (20.1) | <0.001 |
| Peripheral artery disease | 2337 (40.2) | 810 (41.0) | 374 (41.0) | 316 (43.6) | 837 (38.1) | 0.045 |
| Previous stroke/TIA | 706 (12.2) | 182 (9.2) | 103 (11.3) | 99 (13.7) | 322 (14.7) | <0.001 |
| CHA_2_DS_2_-VASc score | 2.7 ± 1.7 | 2.3 ± 1.6 | 2.6 ± 1.7 | 2.9 ± 1.7 | 3.2 ± 1.7 | <0.001 |
| HAS-BLED score | 1.9 ± 1.1 | 1.7 ± 1.1 | 1.8 ± 1.1 | 1.9 ± 1.1 | 2.1 ± 1.1 | <0.001 |
| Chronic kidney disease | 1071 (18.4) | 260 (13.2) | 139 (15.2) | 155 (21.4) | 517 (23.6) | <0.001 |
| Current smoker | 518 (8.9) | 199 (10.1) | 88 (9.6) | 71 (9.8) | 160 (7.3) | 0.009 |
| Regular or social drinker | 1662 (28.6) | 654 (33.1) | 301 (33.0) | 204 (28.1) | 503 (22.9) | <0.001 |
| **Electrocardiography** |  |  |  |  |  |  |
| Heart rate, bpm | 75.7 ±21.8 | 73.8 ±15.8 | 75.2 ±31.9 | 77.9 ±29.2 | 76.9 ±17.9 | <0.001 |
| QRS duration, ms | 99.2 ±24.4 | 99.2 ±19.3 | 98.5 ±17.5 | 100.0 ±42.5 | 99.3 ±22.8 | 0.675 |
| QTc interval, ms | 441.3 ±84.6 | 436.5 ±92.3 | 439.5 ±34.8 | 440.7 ±33.8 | 446.7 ±101.6 | <0.001 |
| Persistent/permanent AF | 1957 (33.7) | 330 (16.7) | 262 (28.7) | 307 (42.3) | 1058 (48.2) | <0.001 |
| Symptomatic AF | 2482 (42.7) | 882 (44.6) | 375 (41.1) | 309 (42.6) | 916 (41.8) | 0.195 |
| **Echocardiography** |  |  |  |  |  |  |
| LVEF, % | 61.6 ± 9.9 | 64.0 ± 8.0 | 62.3 ± 9.2 | 60.7 ±10.1 | 59.4 ±11.1 | <0.001 |
| LVEF < 40% | 263 (4.5) | 26 (1.3) | 30 (3.3) | 39 (5.4) | 168 (7.7) | <0.001 |
| LA diameter, mm | 44.2 ± 8.1 | 37.9 ± 5.3 | 42.8 ± 5.4 | 45.2 ± 5.9 | 50.2 ± 7.1 | <0.001 |
| High LV filling pressure | 1104 (19.0) | 172 (8.7) | 154 (16.9) | 151 (20.8) | 627 (28.6) | <0.001 |

AF, atrial fibrillation; CHF, congestive heart failure; CIED, cardiac implantable electrical device; LA, left atrial; LV, left ventricle;
LVEF, left ventricular ejection fraction; MI, myocardial infarction; RFCA, radiofrequency catheter ablation; TIA, transient ischemic attack.

Data are presented as mean ± standard deviation or number (%).

**Supplemental Table 2. Prescription pattern of medications for atrial fibrillation by left atrial enlargement (by left atrial volume index)**

| **Baseline characteristics** | Overall | No LAE | Mild LAE | Moderate LAE | Severe LAE | P-value |
| --- | --- | --- | --- | --- | --- | --- |
|  | (n=5808) | (n=1977) | (n=912) | (n=725) | (n=2194) |  |
| Anticoagulation | 4147 (71.4) | 1093 (55.3) | 665 (72.9) | 562 (77.5) | 1827 (83.3) | <0.001 |
| Warfarin | 932 (16.0) | 213 (10.8) | 124 (13.6) | 118 (16.3) | 477 (21.7) | <0.001 |
| NOAC | 3357 (57.8) | 920 (46.5) | 558 (61.2) | 462 (63.7) | 1417 (64.6) | <0.001 |
| Antiplatelets | 1330 (22.9) | 562 (28.4) | 215 (23.6) | 161 (22.2) | 392 (17.9) | <0.001 |
| Anti-arrhythmics | 2547 (43.9) | 1111 (56.2) | 461 (50.5) | 273 (37.7) | 702 (32.0) | <0.001 |
| Class I | 1823 (31.4) | 876 (44.3) | 329 (36.1) | 185 (25.5) | 433 (19.7) | <0.001 |
| Class III | 850 (14.6) | 268 (13.6) | 150 (16.4) | 108 (14.9) | 324 (14.8) | 0.230 |
| Others |  |  |  |  |  | <0.001 |
| Beta-blocker | 3026 (52.1) | 981 (49.6) | 472 (51.9) | 378 (52.1) | 1195 (54.5) | 0.020 |
| Calcium channel blocker | 1653 (28.5) | 525 (26.6) | 267 (29.3) | 220 (30.3) | 641 (29.2) | 0.125 |
| Digoxin | 415 (7.1) | 48 (2.4) | 38 (4.2) | 58 (8.0) | 271 (12.4) | <0.001 |

NOAC, non-vitamin K antagonist anticoagulant.

Data are presented as number (%).

**Supplemental Table 3. Clinical outcome according to the degree of left atrial enlargement (by left atrial volume index)**

| **Baseline characteristics** | No LAE | Mild LAE | Moderate LAE | Severe LAE | P-value |
| --- | --- | --- | --- | --- | --- |
| Overall population (N=5808) | (n=1977) | (n=912) | (n=725) | (n=2194) |  |
| Stroke or systemic embolism | 30 (1.5) | 16 (1.8) | 21 (2.9) | 60 (2.7) | 0.021 |
| Death | 21 (1.1) | 12 (1.3) | 11 (1.5) | 32 (1.5) | 0.669 |
| Myocardial infarction | 6 (0.3) | 5 (0.5) | 1 (0.1) | 12 (0.5) | 0.352 |
| MACCE | 56 (2.8) | 30 (3.3) | 32 (4.4) | 101 (4.6) | 0.015 |
| Major bleeding | 13 (0.7) | 11 (1.2) | 3 (0.4) | 24 (1.1) | 0.158 |
| Any bleeding | 119 (6.0) | 79 (8.7) | 63 (8.7) | 171 (7.8) | 0.02 |
| Anticoagulation population (N=4148) | (N=1093) | (N=665) | (N=562) | (N=1827) |  |
| Stroke or systemic embolism | 16 (1.5) | 12 (1.8) | 17 (3.0) | 50 (2.7) | 0.071 |
| Death | 15 (1.4) | 9 (1.4) | 10 (1.8) | 24 (1.3) | 0.873 |
| Myocardial infarction | 4 (0.4) | 3 (0.5) | 0 (0.0) | 10 (0.5) | 0.356 |
| MACCE | 35 (3.2) | 23 (3.5) | 26 (4.6) | 82 (4.5) | 0.259 |
| Major bleeding | 8 (0.7) | 11 (1.7) | 3 (0.5) | 19 (1.0) | 0.173 |
| Any bleeding | 89 (8.1) | 68 (10.2) | 54 (9.6) | 140 (7.7) | 0.153 |

MACCE, major adverse cardiac and cerebrovascular event.

Data are presented as number (%).

**Supplemental Table 4. Prognostic implication of significant left atrial enlargement (by left atrial volume index) on the clinical outcomes**

|  | Crude analysis | | | | Multivariable analysis | |
| --- | --- | --- | --- | --- | --- | --- |
|  | Event rate (%) at 2 years | |  |  |  |  |
|  | No or mild LAE | Moderate-severe LAE | HR (95% CI) | P-value | HR (95% CI) | P-value |
| Overall population (N=5808) | (n=2919) | (N=2889) |  |  |  |  |
| Stroke or systemic embolism | 1.4 | 2.5 | 1.81 (1.26–2.60) | 0.001 | 1.58 (1.09–2.29) | 0.017 |
| Death | 1.4 | 1.1 | 1.33 (0.85-2.10) | 0.214 | 1.03 (0.65-1.65) | 0.899 |
| Myocardial infarction | 0.4 | 0.4 | 1.22 (0.55-2.72) | 0.628 | 0.98 (0.43-2.25) | 0.959 |
| MACCE | 2.6 | 4.2 | 1.59 (1.22-2.09) | <0.001 | 1.34 (1.01-1.77) | 0.043 |
| Major bleeding | 0.8 | 0.9 | 1.15 (0.67-2.00 | 0.610 | .097 (0.55-1.71) | 0.916 |
| Any bleeding | 6.2 | 7.6 | 1.23 (1.01-1.48) | 0.035 | 1.05 (0.86-1.27) | 0.661 |
| Anticoagulation population (n=4147) | (n=1758) | (n=2389) |  |  |  |  |
| Stroke or systemic embolism | 1.4 | 2.6 | 1.83 (1.18-2.85) | 0.007 | 1.72 (1.10-2.71) | 0.018 |
| Death | 1.3 | 1.4 | 1.08 (0.64-1.82) | 0.773 | 1.01 (0.59-1.72) | 0.984 |
| Myocardial infarction | 0.4 | 0.5 | 1.10 (0.42-2.88) | 0.850 | 1.00 (0.37-2.72) | 0.995 |
| MACCE | 3.0 | 4.3 | 1.43 (1.04-1.97) | 0.029 | 1.35 (0.97-1.87) | 0.072 |
| Major bleeding | 1.1 | 1.0 | 0.89 (0.48-1.64) | 0.702 | 0.86 (0.46-1.62) | 0.649 |
| Any bleeding | 8.3 | 7.9 | 0.94 (0.76-1.16) | 0.580 | 0.91 (0.73-1.13) | 0.372 |

MACCE, major adverse cardiac and cerebrovascular event.

*Adjusted for age, sex, hypertension, diabetes, previous stroke, heart failure, and vascular disease.

**Supplemental figure 1.** Side-by-side demonstration of rate of stroke or systemic embolism according to the presence of significant left atrial enlargement by left atrial volume index or left atrial anterior-posterior dimension


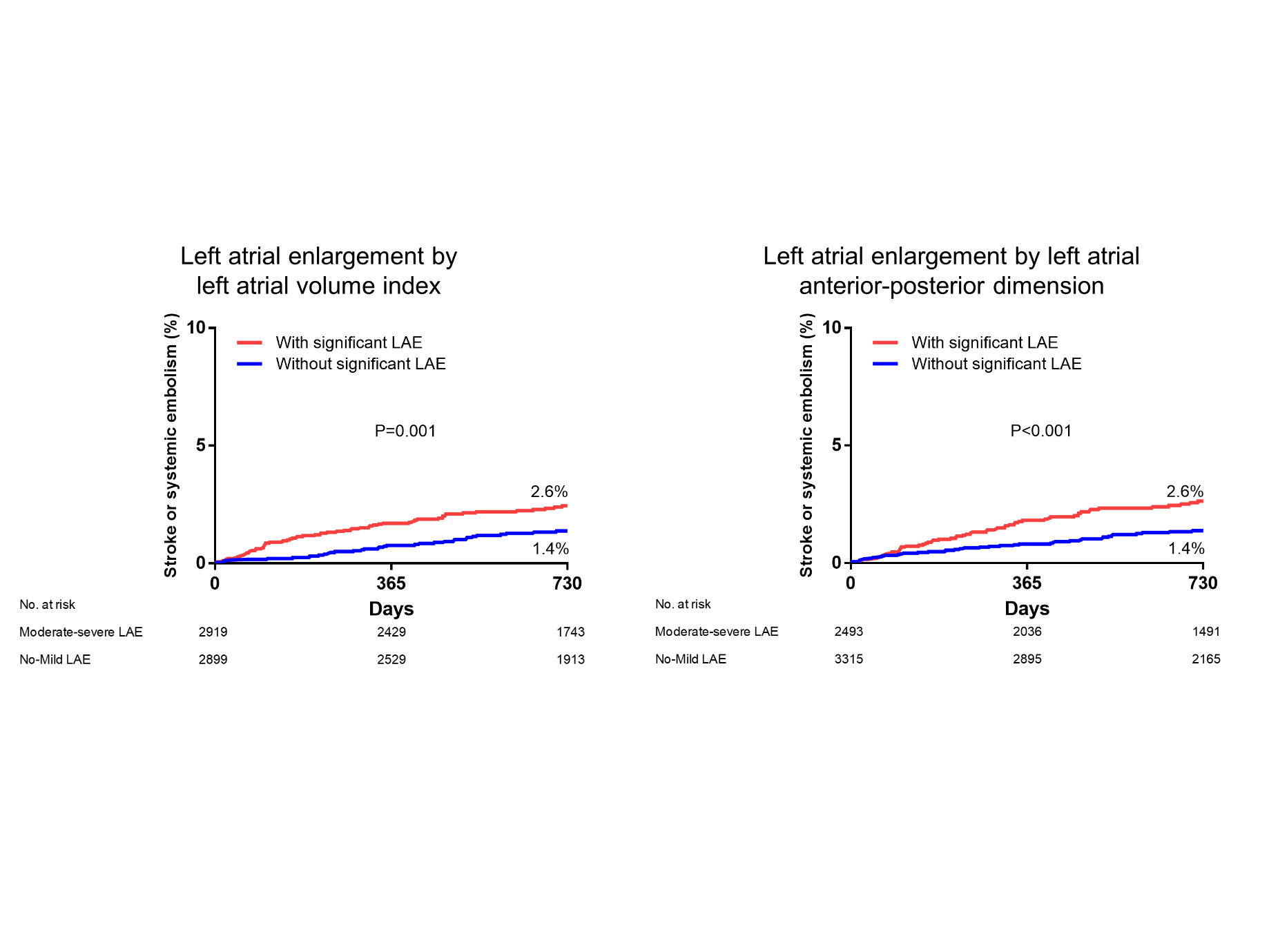


**Supplemental figure 2.** Side-by-side demonstration of rate of stroke or systemic embolism after use of non vitamin K antagonis oral anticoagulants or warfarin in patients with significant left atrial enlargement by left atrial volume index or left atrial anterior-posterior dimension


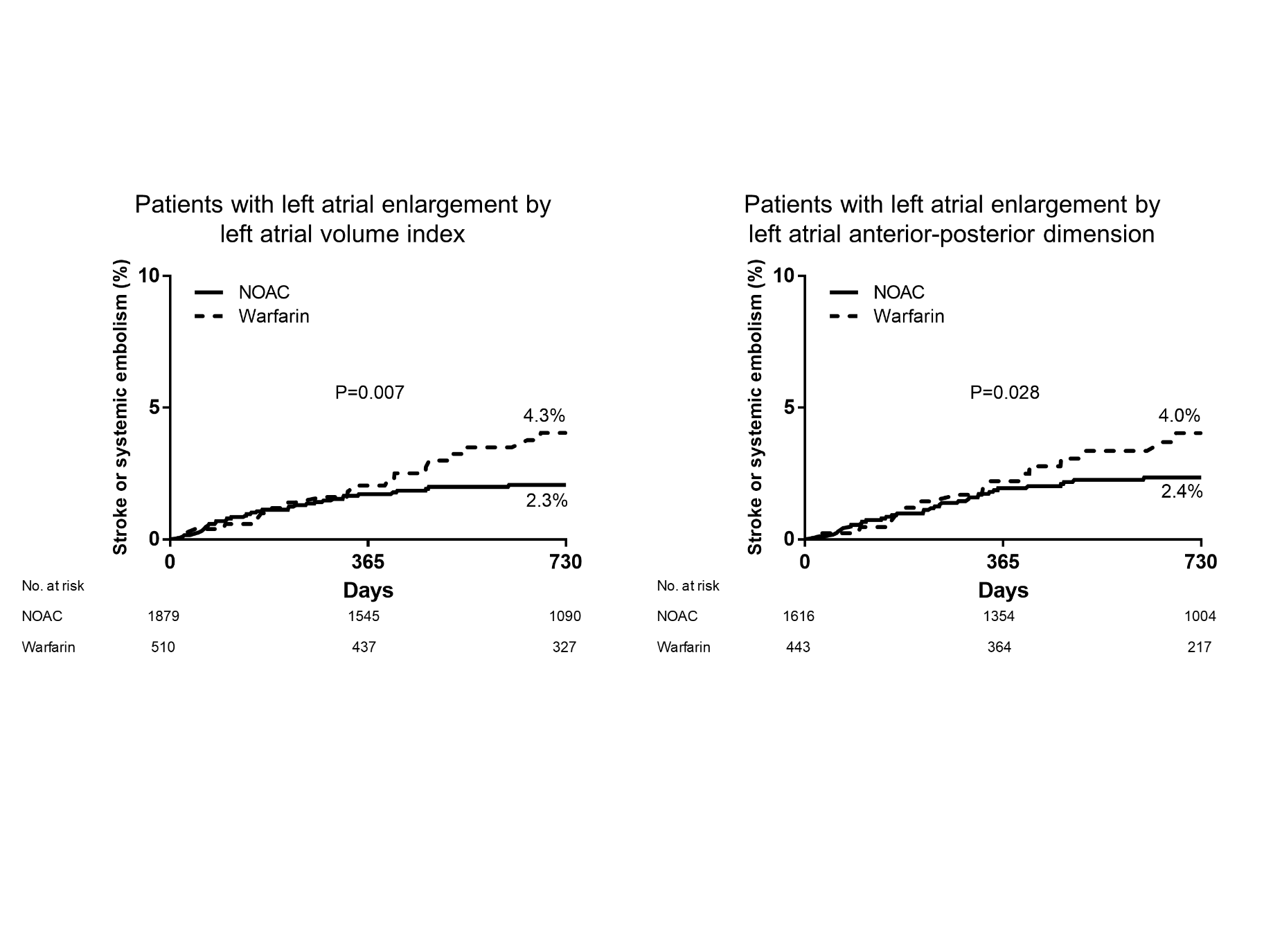

Supplement: Supplementary file 2 — Supplementary Information 2. [file 41598_2021_3266_MOESM2_ESM.docx]
